# Synthesis of Trigeneration Systems: Sensitivity Analyses and Resilience

**DOI:** 10.1155/2013/604852

**Published:** 2013-12-25

**Authors:** Monica Carvalho, Miguel A. Lozano, José Ramos, Luis M. Serra

**Affiliations:** ^1^Department of Renewable Energy Engineering, Center of Alternative and Renewable Energy, Federal University of Paraíba, Campus I, 58059-970 João Pessoa, PB, Brazil; ^2^Group of Thermal Engineering and Energy Systems (GITSE), Aragon Institute of Engineering Research (I3A), Universidad de Zaragoza, Campus Rio Ebro, 50018 Zaragoza, Spain

## Abstract

This paper presents sensitivity and resilience analyses for a trigeneration system designed for a hospital. The following information is utilized to formulate an integer linear programming model: (1) energy service demands of the hospital, (2) technical and economical characteristics of the potential technologies for installation, (3) prices of the available utilities interchanged, and (4) financial parameters of the project. The solution of the model, minimizing the annual total cost, provides the optimal configuration of the system (technologies installed and number of pieces of equipment) and the optimal operation mode (operational load of equipment, interchange of utilities with the environment, convenience of wasting cogenerated heat, etc.) at each temporal interval defining the demand. The broad range of technical, economic, and institutional uncertainties throughout the life cycle of energy supply systems for buildings makes it necessary to delve more deeply into the fundamental properties of resilient systems: feasibility, flexibility and robustness. The resilience of the obtained solution is tested by varying, within reasonable limits, selected parameters: energy demand, amortization and maintenance factor, natural gas price, self-consumption of electricity, and time-of-delivery feed-in tariffs.

## 1. Introduction

Presently, energy consumption of buildings in developed countries comprises 20–40% of total energy use and is greater than industry and transport figures in the European Union (EU) and USA [1].

**Figure 1:**
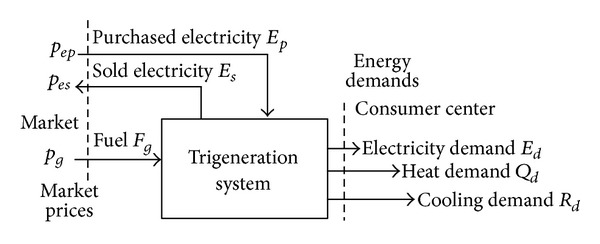
Trigeneration system.

A trigeneration system (Figure 1) is designed with the purpose of satisfying the predictable demands of electricity, heat for heating and sanitary hot water (SHW), and cooling of a given consumer center. The technology behind trigeneration is fundamentally based on the coupling of a cogeneration module with an absorption chiller. The cogeneration module includes a thermal motor (a gas turbine or reciprocating engine, e.g.) that converts the fuel's energy into mechanical energy, an alternator that converts the mechanical energy into electrical energy, and a set of heat exchangers to recuperate useful heat. The absorption chiller can produce cooling by means of using recuperated heat. There are different types of trigeneration systems, which are distinguished by the incorporation of additional equipment [2–4]. Usually, the trigeneration system is complemented by hot water or steam boilers and mechanical chillers. Both technologies are used to guarantee supply and also to avoid oversizing the cogeneration module and the associated absorption chiller. The key in operating trigeneration systems is to satisfy the energy demands of the consumer center with a minimal economic cost. So although a trigeneration system can be designed to operate independently from the electric grid, it is beneficial to establish a link with the grid to buy and sell electricity.

The enhanced fuel consumption efficiency, thanks to the energy integration of the processes in its equipment, is the main benefit of the production of three energy services (electricity, heat, and cooling) from the same energy source in an optimized trigeneration system [5, 6]. This better use of fuel resources is important, as it is associated with economic savings and sparing of the environment with less pollution generated. In order to maximize these benefits, the optimal design of trigeneration plants for buildings needs to address two fundamental issues: the synthesis of the plant configuration and operational planning. Daily and seasonal variations of heating and cooling demands are factors that exert the most influence on the appropriate structure of the energy supply system. A structure can only be selected when consideration is given to the optimal operation of the system's different components on an hour-by-hour basis throughout the year, which in turn depends on energy market prices.

Many different feasible configurations with different operation modes are involved in an analysis, thus resulting in a complex and difficult problem to solve. The review of Chicco and Mancarella [7] summarizes the characteristics of the optimization methods for polygeneration systems. Developing a custom-build model including different technologies, unit sizes, control modes, market, and legal restrictions can be a complex and laborious process, but the end result will be more transparent to their users. Its principal advantage is that it can be easily modified to respond to different scenarios [8].

The actual development of sustainable energy systems remains challenging due to the broad range of technical, economic and institutional factors that need to be considered throughout the system life cycle [9]. It is necessary to delve more deeply into the fundamental properties of successful systems: efficiency, flexibility, and robustness, rather than utilizing traditional methods for analyzing cost, benefits, and risk. The design of an energy production system should consider not only the technical, but also the economic and institutional uncertainties [10]. By uncertainty, we mean the general lack of knowledge about how the future will unfold.

Sensitivity analysis is the study of how the variation in the output of a model can be apportioned, qualitatively or quantitatively, to different sources of variation, and, or how the given model depends upon the information fed into it [11, 12]. Sensitivity analysis is a technique used to determine how different values of an independent variable will impact a particular dependent variable under a given set of assumptions. Sensitivity analysis is very useful when attempting to determine the impact the actual outcome of a particular variable will have if it differs from what was previously assumed. By creating a given set of scenarios, the analyst can determine how changes in one variable will impact the target variable.

Uncertainty issues become more and more important for the long term planning of energy supply systems for buildings. Optimal designs of small cogeneration plants in a market with fluctuating electricity prices are presented and discussed by Lund and Andersen [13]. Basulto Ernst and Perrella Balestieri [14] proposed an approach to model thermal and electric load variability in order to evaluate the effects of load fluctuation on the cogeneration plant design. The influence of the life of a fuel cell stack due to performance degradation on a system's economy was investigated by Ito et al. [15]. Gamou et al. investigated the influences of tolerating the shortage of energy supplies on the economy of cogeneration systems and optimal equipment capacities [16] and proposed an optimal unit sizing method for cogeneration systems taking into consideration the uncertainty of energy demands as continuous random variables [17].

In the paper of Yoshida et al. [18] the best system structure and operational strategy is determined for the energy supply system for a hospital. Sensitivity analysis is carried out to verify the influence of upgrading the performance of equipment (+5% to +10%), initial capital cost of equipment (−20% to −50%), and price cutting charges of natural gas and electricity (−5% to −10%). Ren and Gao [19] conducted an investigation on the optimal energy system plan for an ecocampus in Kitakyushu, Japan.

The authors of the two aforementioned works [18, 19] have extended existing studies a step further. These works not only account for the increase/decrease in capacity of installed equipment but also note that some equipment may disappear or appear and that the proposed equipment is not of continuous capacity but actually commercially available with specific dimensions and technical characteristics. A similar sensitivity analysis was also carried out by us in two previous papers. In the first paper [20], the effect of the annual amortization factor and maintenance factor fam (−50% to +50%) and natural gas price (−40% to +40%) were verified on the energy supply system of a hospital (same system analyzed in the present paper). In the second paper [21], the effect of these two parameters was analyzed, fam (−25% to +100%) and natural gas price (−30% to +40%), in addition to the effect of legal restrictions on the sale price of electricity and on minimum self-consumption. The advantages of including heat and cold storage were also added to the study.

Although the concepts of resilience and robustness are not novel, the number of papers centered exclusively on the aspect of evaluating resilience or robustness of energy systems is very limited. Morari [22] included aspects of flexibility, operability, and control into the design procedures of a chemical plant. Larsson and Wene [23] presented a method using hedging to carry out a quantitative analysis of the robustness of the Swedish energy system, further applying the method to evaluate the efficiency and robustness of three strategies [24]. Lai and Hui [25] studied flexibility and feasibility characteristics of a trigeneration system with a predefined structure to handle periodical demand deviations.

At this point, we will discuss the innovations that will be added to our research to even further expand the existing knowledge base. Firstly, the robustness of the optimal structure corresponding to the adopted design will be analyzed. In other words, it will be investigated how a concrete design preestablished as an optimal configuration will support external changes. The analysis will not consider the validity or quality of the mathematical model employed but will detail how the optimization decision supports future technical and economic uncertainties (i.e., energy demands, amortization and maintenance costs, and gas prices). Secondly, an approach for resolution of legal constraints (minimum self-consumed electricity, type of feed-in tariff) will be discussed. To the best of our knowledge and based on systematic reviews, no similar studies have been conducted for comparison purposes.

In a previous paper [20] the authors provided detailed information on energy service demands for a 500-bed hospital located in Zaragoza (Spain), explained the superstructure of the energy supply system considered for the synthesis, and developed a mixed integer linear programming (MILP) model for the multiperiod synthesis and operational planning problem, including (1) the determination of the type, number, and capacity of the equipment installed in the energy supply system and (2) the establishment of the optimal operation for the different plant components on an hourly basis throughout a representative year. Now, in this paper, we analyze how to use the mathematical programming model to provide decision makers with information on the underlying decision problems. This paper does not present a new methodology; this paper carries out sensitivity and resilience analyses. They are applied to the configuration of a system with fixed-size commercially available equipment (discrete design variables, not continuous). An insight into a solution reveals how optimal decisions are affected by information updates on demand variations, economic factors, and legal constraints, that is, the degree of resilience of the optimal solution obtained.

## 2. Description of the Optimal Trigeneration System

Health care is an energy-intensive, energy-dependent enterprise. Hospital facilities are different from other buildings in that they operate 24 h/day year-round and require sophisticated backup systems in case of utility shutdowns. Hospitals are good candidates for trigeneration systems because of their high energy requirements compared to other commercial buildings as well as their need for high power quality and reliability [26].

A previous paper [20] provided detailed information on energy service demands for the hospital, explained the superstructure of the energy supply system considered for the synthesis of the trigeneration system (available technologies as well as technical and economic characteristics of equipment and operation modes), and presented energy purchase/sale tariffs and current legal requirements for operating a cogeneration system in Spain. Regarding the economic objective function, it was observed that the installation of energy-efficient technologies (cogeneration modules and absorption chillers) was beneficial to achieve the minimum annual cost.

### 2.1. Energy Demand

The energy demands considered for the hospital were heat, cooling, and electricity. The heat load included heat for sanitary hot water and for space heating. In order to model the energy demands, a study period of one year was considered, distributed in 24 representative days (one working day and one holiday/weekend day for each month), each day being divided into 24 hourly periods. The annual electricity consumption of the hospital was 3250 MWh, the cooling demand was 1265 MWh, and the heat requirements (SHW + heating) were 8059 MWh.

### 2.2. System Configuration

**Figure 2:**
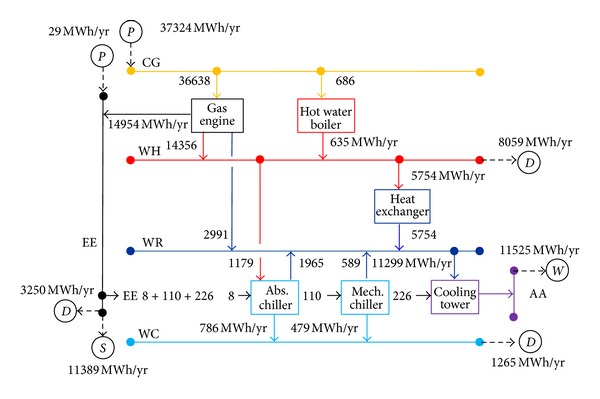
Structure and annual operation of the optimal trigeneration system.

The superstructure of the energy supply system for the hospital considered the possibility of installing energy production technologies such as gas turbine, steam boiler, internal combustion gas engine, hot water boiler, heat exchangers (steam-hot water and hot water-cooling water), double and single effect absorption chillers, mechanical chiller, and cooling tower. All technology and equipment considered in the optimization were commercially available and therefore the size/configuration of the system was determined in terms of pieces of equipment. The optimal solutions obtained in Lozano et al. [20] only present the following technologies: MGWH (internal combustion gas engine + hot water heat recovery system), CGWH (hot water boiler), ICWH (hot water-cooling water heat exchanger), FAWH (single effect absorption chiller, driven by hot water), FMWR (mechanical chiller, driven by electricity and cooled by water), and CTWR (cooling tower, to evacuate the heat from the cooling water to the ambient air).  Figure 2 depicts the structure of the optimal economic energy supply system, showing the technologies selected, the present energy utilities, and the interactions between technologies and utilities. The present utilities are: CG (natural gas), WH (hot water, 90°C), WR (cooling water, *t*0 + 5°C), AA (ambient air, temperature *t*0 in Celsius), WC (chilled water, 5°C), and EE (electricity). D, S, P and W refer to, respectively, demand, sale, purchase and waste/loss of a utility.

Table 1 depicts the selected equipment and technical production coefficients. The rows contain installed technologies and the columns contain the utilities. *P*_nom⁡_ is the nominal power of the equipment. The production coefficient with a bold 1 shows the flow that defines the equipment's capacity. Positive coefficients indicate that the utility is produced, while negative coefficients indicate the consumption of such utility. It was considered that the production coefficients were constant and independent from the production *P* ≤ *P*_nom⁡_ of the equipment at a given moment. The data shown in Table 1 was obtained from equipment catalogs and consultations with manufacturers.

ZI(*i*) in Table 1 is the total investment cost of the selected equipment of technology *i*, obtained from the catalog price and multiplied by two factors: (1) a simple module factor that took into account transportation, installation, connection, insulation, and so forth [27–30] and (2) a factor of indirect costs, which includes engineering and supervision expenses, legal expenses, contractor's fees, and contingencies (equal to 15% of the equipment investment costs).

Considering the lifetime of the plant to be 15 years and an interest rate of 0.10 yr^−1^ (reasonable for the present economic circumstances in Spain), an annual amortization factor of 0.13 yr^−1^ was obtained. Annual maintenance and operating costs, different from energy costs, were considered to be 7% of the total investment cost. The factor fam = 0.20 yr^−1^ took into account both amortization and maintenance factors.

### 2.3. Energy Prices and Regulation

A purchase cost of *p*_*g*_ = 25 €/MWh for natural gas, which includes taxes and the distribution of fixed costs throughout the estimated annual consumption, was considered [20]. Considering other costs such as taxes, and approximating the distribution of fixed costs, an electricity purchase price of *p*_ep_ = 95 €/MWh for off-peak hours and *p*_ep_ = 130.15 €/MWh for on-peak hours was considered [20].

According to Spanish legislation and accounting for the nominal power of the natural gas cogeneration modules, the price for sold electricity was *p*_es_ = 77 €/MWh. In this case it is advisable to verify if the system is capable of maintaining an equivalent electrical efficiency, calculated on an annual basis, of at least 55% for internal combustion gas engines [20].

### 2.4. Optimization Model

An optimization model was built based on mixed integer linear programming and its solution provides the most convenient configuration and operation modes. The objective function considered is the annual total cost *C*_tot_ (in €/yr):

(1) $$\begin{array}{c} {\mathrm{Min}C}_{\text{tot}}=C_{\text{fix}}+C_{\text{ope}}, \end{array}$$

which minimized equipment and fuel costs as well as purchase/sale of energy services. The annual fixed cost of the equipment *C*_fix_ was expressed by

(2) Cfix=∑iCfix(i)=fam·∑iZ(i)=fam·∑iNIN(i)·ZI(i),

where NIN(*i*) and ZI(*i*) were, respectively, the number of pieces of equipment installed and the capital cost of each piece of equipment installed for technology *i*.

Considering that the year was divided into days, which were in turn subdivided into hours, (*d*, *h*) represented the *h*th hour of the *d*th day. The annual energy cost *C*_ope_ associated with the operation of the system was expressed by

(3) Cope=∑d ∑h[pg·Fg(d,h)+pep(d,h)·Ep(d,h)−pes(d,h)·Es(d,h)],

where energy flows are expressed in MW. *F*_*g*_ was the consumption of natural gas, and *E*_*p*_ and *E*_*s*_ were the amount of electricity purchased and sold, respectively.

Operation was subject to capacity limits, production restrictions, and balance equations.

The MILP model for the multiperiod synthesis and operational planning problem was characterized by integer variables for the determination of the number of units installed, and by continuous variables for the representation of energy and economic flows and funds. MILP techniques, already applied in the optimization of cogeneration and trigeneration systems by several authors [31–36], were used. More details on the optimization model can be found in Lozano et al. [20]. The model was implemented in the LINGO [37] modeling language and optimizer, a commercial software package for solving optimization problems. The optimal solution was a global optimal which was obtained by the optimization model through an implicit comparison of the annual optimal operation of all possible structures.

### 2.5. Legal Conditions

The primary energy savings (PES) provided by cogeneration were calculated in accordance with

(4) $$\begin{array}{c} \text{PES}=100\cdot \left[1-\frac{F_{c}}{\left(E_{c}/\eta_{\text{ec}}+Q_{\text{cc}}/\eta_{\text{qc}}\right)}\right], \end{array}$$

where *η*_ec_ = 0.48 and *η*_qc_ = 0.90 are the efficiency reference values given in the EU's commission decision [38] for the separate production of electricity and heat, respectively. *E*_*c*_ is the cogenerated electricity, *F*_*c*_ is the consumption of natural gas measured by its lower heating value (LHV), and *Q*_cc_ is the cogenerated useful heat.

A legal condition is applied when cogeneration modules are installed specifying that cogeneration systems must have an annual equivalent electrical efficiency (EEE) value, defined as

(5) $$\begin{array}{c} \text{EEE}=100\cdot \frac{E_{c}}{\left(F_{c}-Q_{\text{cc}}/\eta_{\text{qc}}\right)}, \end{array}$$

higher than 55%, which is the minimum required by Spanish law for electricity production in a Special Regime [39] when natural gas-fired reciprocating engines are installed.

### 2.6. Optimal Trigeneration and Conventional Systems

Table 2 displays the structure of the system and relevant annual energy and monetary flows for the optimal economic system. The economic optimization suggested the installation of three cogeneration modules, three hot water boilers, one absorption chiller, three mechanical chillers, four hot water-cooling water heat exchangers, and three cooling towers, as shown in Figure 2.

Electricity was supplied to users by operating gas engine cogeneration modules and by purchasing a small quantity from an outside electric power company. Electricity was used to drive the mechanical chillers and auxiliary machinery in this system. Hot water for SHW, space heating, and to drive the single effect absorption chiller was supplied by the cogeneration modules and gas-fired boilers. Surplus not-consumed cogenerated heat was disposed of through ICWH hot water-cooling water heat exchangers. Cold water for space cooling was supplied by the single effect absorption chiller and mechanical chillers. The system took advantage of the low purchase cost of natural gas and achieved profit by selling as much as possible cogenerated electricity to the electric grid. There is a great amount of heat being wasted into the environment. Note that the equivalent electrical efficiency of the optimal solution is 55%, the lowest amount allowed.

It is worthwhile highlighting the fact that the optimization results shown herein are presented in annual form. As the electricity tariffs vary throughout the day and throughout the year, at some specific times it is advantageous for the energy supply system to sell self-generated electricity to the grid (taking advantage of the high price for electricity export) while purchasing electricity to the grid. Constraints in the optimization model (please refer to [20] for details) prevent the system from importing and exporting electricity from/to the grid simultaneously (the system cannot purchase electricity from the grid and then sell it back).

**Figure 3:**
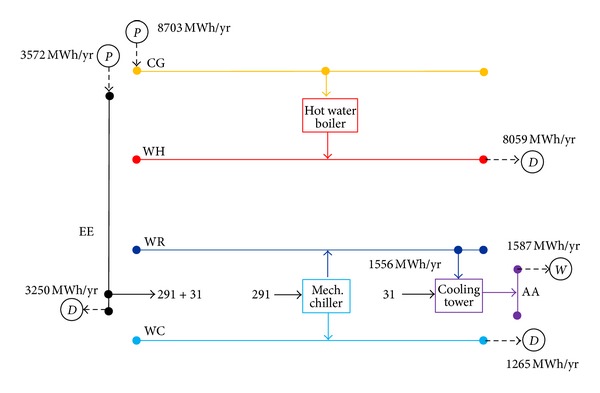
Structure and annual operation of the conventional system.

The characteristics of the conventional energy supply system were obtained when excluding the possibility of cogeneration in the optimization model. The configuration and main energy flows are shown in Figure 3. Electricity was purchased directly from the grid to meet the demands of electricity and cooling through four mechanical chillers and three cooling towers. Heat was produced by six hot water boilers. Table 2 displays the structure of the system and relevant energy and monetary flows for the conventional system.

The annual energy cost savings achieved with the trigeneration system compared to the conventional system are 525 195 €/yr. The installation of a trigeneration system requires an additional investment, compared with the conventional system, of 1 455 900 €/yr, resulting in a simple payback period of less than three years.

## 3. Energy Demands

The first traditional analyses study the effects of the variation of energy demands. In these first analyses, there is total freedom in the optimization model, which will result in different configurations with different operation strategies.

The energy demands were varied within the range −20% to +20% in 5% steps.  Table 3 displays the type and number of installed equipment and annual energy and monetary flows for the optimal solutions. The column in bold indicates the base case.

As the energy demands decreased from the base case, the number of cogeneration modules and the purchase of natural gas decreased. As the energy demands increased from the base case, the basic configuration with three gas engines and one absorption chiller remained constant, but cooling towers, mechanical chillers, and hot water boilers are added.

The solutions presented in Table 3 display the desirable feature of flexibility, as all solutions not only matched the energy demands of the consumer center, but did also so in an efficient and profitable manner. The term flexibility will be used here to describe more than a condition of the system, but a virtue, as the system adapts to different conditions in the environment. This concept takes the definition of feasibility a step further, not only considering that the energy demands of the consumer center were satisfied, but that the system adapted well, economically and efficiently, to the changes. The concept of flexibility expresses a leap between the reference system (that satisfactorily meets the energy demands) and the proposed system with several equipment integrated, which is able to operate in different operational states and with economic benefits.

Previous traditional sensitivity analyses considered the design of a new system with total freedom in the optimization model. However, in real projects, a robust system configuration is desired, with adequate objective function values valid not only at the optimal calculated, but also when there are changes in boundary conditions of parameters. If the configuration obtained is sensitive to small perturbations, it will not be appropriate because the high investment will result in serious risk in practice. So, the second set of analyses will verify the robustness of the optimal economic configuration presented in Table 2 (base case). From an entrepreneurial viewpoint, it will be verified how the base case system will operate when a change in conditions is implemented. The concept of robustness here expresses that the configuration not only fulfils the series of initial conditions in an economic and efficient way, but also in the event of unexpected changes, the system adapts well and still delivers an economic and efficient operation. Robustness analyses are presented as a first approach for dealing with an increasingly common problem, unpredictably changing environment, and how the system adapts to such changing conditions.

The term resilience expresses the capability of the system of adapting to expected changes (flexibility) as well as to unexpected changes (robustness). So, the concept of resilience will encompass the previous concepts of feasibility, flexibility, and robustness of the base case configuration. It will be considered that the system has already been built, and the question is how the system will react to future unforeseeable changes in external conditions. In this situation, only an operational retrofit will take place. The optimization model verifying the resilience will consider a fixed configuration, optimizing the operational strategy throughout the entire year when the energy demands vary between the intervals previously specified.

The starting point to these analyses is the base case configuration, with three gas engines, three hot water boilers, four hot water-cooling water heat exchangers, one absorption chiller, three mechanical chillers, and three cooling towers (i.e., the optimal economic configuration obtained in Section 2).  Table 4 shows the results for the operational optimal strategy considering the fixed configuration and that only the energy demands varied.

For variations of over 10% in the energy demands, the solution with the base case configuration was unfeasible, as obviously the existing installed equipment was not sufficient to satisfy the increased energy demands. However, from Table 3 it can be seen that when the energy demands increase, the configuration of the system presents a modular behavior in the sense that only conventional (and cheaper) equipment is added. Therefore, in the event that energy demands increase more than 10%, hot water boilers, mechanical chillers and cooling towers are added to guarantee the supply of the consumer center.

The data shown in Table 4 suggests that the configuration of the base case was reasonable in terms of robustness against perturbations of demand. Variation of energy demands was absorbed by the system, which adapted its operational mode to the new scenarios, even with an increase in energy demands over 10%.

Tables 3 and 4 present different ranges for variations in energy demands. This occurs because Table 3 presents a sensitivity analysis that allows structural changes to adapt to unlimited changes in energy demands.  Table 4, on the other hand, studies the resilience of a selected structure (fixed), which is analyzed until the energy demand limit that can be satisfied without incorporation of new equipment.

**Figure 4:**
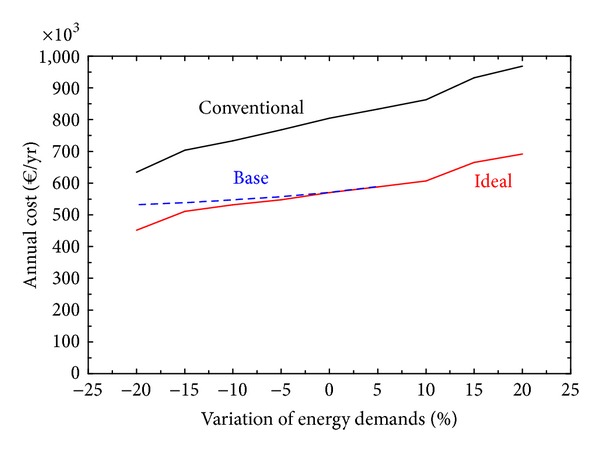
Behavior of solutions in response to variations in energy demands.

Figure 4 shows the behavior of three solutions in response to variations in energy demands: Conventional, Ideal, and Base. The Conventional solution meets the energy demands of the hospital through the purchase of natural gas for the boilers, which will satisfy the heat demand and through the purchase of electricity from the electric grid to satisfy the demands of electricity and cooling (via mechanical chillers). The Base solution is the optimal solution corresponding to a system with the base case configuration (data in Table 4). The Ideal solution corresponds to the optimal solution obtained with free selection of technologies (data in Table 3).

From Figure 4 it can be seen that the base case design can be considered a good selection, behaving very closely to the optimal ideal solutions (with free choice of equipment) in an interval of energy demands variation between −15% and +5%. When the energy demands vary up to −15%, the maximum difference between the total annual costs of Base and the Ideal configurations is 5.45%, which is quite acceptable. In this way, the base case design displayed a resilient behavior, delivering good results even when confronted with unpredictable changes in demand conditions.

## 4. Economic Factors

The following analyses study the effects of the variation of two economic factors: amortization and maintenance factor and price of natural gas. The amortization and maintenance factor weighs capital costs in comparison with energy costs, and depends on the lifetime of the systems, the interest rate, and maintenance and operating cost. Variation of the price of natural gas will vary the relationship with the price of electricity, so the analysis also covers indirectly variations in the price of electricity.

### 4.1. Amortization and Maintenance Factor

The first analyses are carried out in the traditional sense, with free configuration and operation of the system in the optimization model. The influence of the amortization and maintenance factor fam was analyzed, varying between 0.10 and 0.30 yr^−1^.  Table 5 displays the type and number of installed equipment and annual energy and monetary flows for the optimal design. The column in bold indicates the base case.

A trend was observed: as the fam factor increased, the number of cogeneration modules and absorption chillers as well as the sale of electricity decreased. There is no purchase of electricity with fam less than 0.20 yr^−1^, when three gas engines and two absorption chillers were installed. With fam = 0.20 yr^−1^, one absorption chiller with one cooling tower was replaced by one mechanical chiller, reducing the investment. With fam greater than 0.20 yr^−1^ a gas engine was eliminated, reducing the inversion but with a consequent reduction in the production of electricity and cogenerated heat. The sale of electricity decreased and it was necessary to install another hot water boiler to supply heat.

Following the methodology, flexibility analyses followed by robustness analyses, now we will verify the robustness of the base case configuration. It will be considered that the operational retrofit is forced onto the base case system due to changes in fam and verified whether the base case configuration is able to tackle unexpected conditions, resulting in a resilient configuration.  Table 6 shows the results for the operational optimal strategy considering the base case configuration when fam varied.

The data shown in Table 6 suggests that the base case configuration operated correctly across a wide range of fam conditions. Logically, variation of fam did not affect the operational strategy of the system.

**Figure 5:**
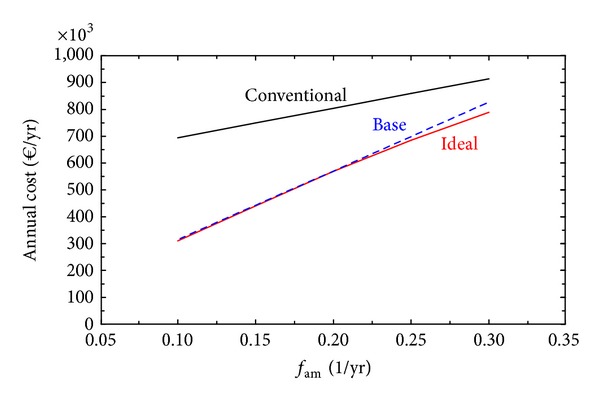
Behavior of solutions in response to variations in fam.

Figure 5 shows the behavior of three solutions in response to variations in fam: Conventional, Base, and Ideal. The conventional solution meets the energy demands of the hospital through the purchase of natural gas for the boilers, which will satisfy the heat demand and through the purchase of electricity from the electric grid to satisfy the demands of electricity and cooling (via mechanical chillers). The base case configuration is the fixed configuration corresponding to the initial economic optimal (data in Table 6). The ideal solution corresponds to the free selection of technologies (data in Table 5). Figure 5 revealed that the base case design was a wise selection, being stable for a wide interval of fam and behaving closely to the optimal ideal solutions (with free choice of equipment), resulting in a resilient configuration.

### 4.2. Price of Natural Gas

Secondly, the influence of the natural gas price was analyzed.  Table 7 displays the type and number of installed equipment for values of *p*_*g*_ between 0.015 and 0.035 €/kWh and annual energy and monetary flows for the economic optimal. The column in bold indicates the base case.

As the price of natural gas increased, the number of cogeneration modules and absorption chillers as well as the sale of electricity decreased. There is no purchase of electricity with *p*_*g*_ less than 0.025 €/kWh, when three gas engines and two absorption chillers were installed. With *p*_*g*_ = 0.025 €/kWh, one absorption chiller and one cooling tower were replaced by one mechanical chiller. This reduced the investment but required purchasing electricity externally. With *p*_*g*_ = 0.030 €/kWh, one gas engine was eliminated, reducing the inversion but with a consequent reduction in the production of electricity and cogenerated heat. The sale of electricity decreased and it was necessary to install another hot water boiler to supply heat. With *p*_*g*_ = 0.035 €/kWh, only one gas engine was installed, the sale of electricity decreased to a reduced value and it was necessary to install another hot water boiler to supply heat. Not much variety was observed in the optimal configurations, and the results were logical in the sense that the operation of the system adapted to the price of natural gas, realizing profit by taking advantage of its low price and selling electricity to the grid.

Following the proposed methodology, now the robustness of the base configuration will be verified. It will be considered that the operational retrofit is forced onto the system due to changes in the price of natural gas and verified whether the base case configuration is able to tackle unexpected conditions, resulting in a resilient configuration.  Table 8 shows the results for the operational optimal strategy considering the base case configuration when the price of natural gas varied.

The data shown in Table 8 suggests that the base configuration was good in terms of optimality and also good in terms of robustness against perturbations. Variations in operation occurred only for high prices (*p*_*g*_ = 0.035 €/kWh), when less natural gas was purchased (and consequently less cogenerated electricity was sold to the grid).

**Figure 6:**
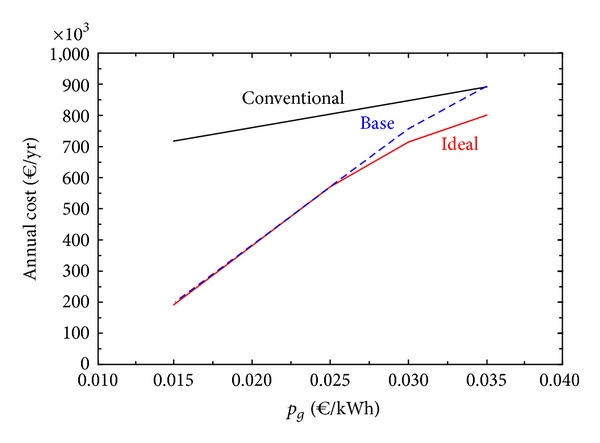
Behavior of solutions in response to variations in the price of natural gas.

Figure 6 shows the behavior of three solutions in response to variations in the price of natural gas: Conventional, Base, and Ideal. The base case configuration is the fixed configuration corresponding to the initial economic optimal (data in Table 8). The ideal solution corresponds to the free selection of technologies (data in Table 7).

Figure 6 revealed that the base case design was an appropriate selection, behaving closely to the ideal solutions (with free choice of equipment). Only with high natural gas prices, both the base and ideal solutions are not much better than the conventional solution.

In many practical optimization tasks, there is a need to search for robust plants with adequate configurations to withstand perturbations of market conditions without significant loss of economic performance. With respect to the economic parameters, Figures 5 and 6 revealed that the base case design was resilient to variations of annual amortization and maintenance factor and natural gas price, behaving closely to the optimal ideal solutions (with free choice of equipment). This means that investing in the base case system is convenient, with the exception of a dramatic change in the economic environment.

## 5. Legal Constraints

In Spain, electricity producers in the Special Regime (those that use cogeneration, renewable sources, and waste products) can sell their surplus electricity at a regulated tariff. Cogeneration plants are restricted basically by the following legal constraints: (i) electric power must be lower than 50 MW; (ii) a minimum equivalent electric efficiency must be fulfilled, depending on the cogeneration technology and the fuel consumed; and (iii) a specific quota of the electricity produced must be self-consumed by the owner of the cogeneration plant. During the last years, legal restrictions have been modified [40]. The following analyses verify the effect of legal constraints in Spain regarding minimum self-consumption and time-of-delivery feed-in tariffs on the optimal economic energy supply system. An additional legal condition for all scenarios is an annual equivalent electrical efficiency value higher than 55%, which is the minimum required by Spanish law when cogeneration modules with natural gas-fired reciprocating engines are installed. The amortization and maintenance factor fam and prices of natural gas and purchased electricity were the same for all scenarios (fam = 0.20 yr^−1^, *p*_*g*_ = 0.025 €/kWh, *p*_ep_ = 0.095 €/kWh).

### 5.1. Self-Consumption

In recent years, legal restrictions have been modified and the most significant difference has been the mandatory minimum amount of self-consumed electricity. In 1998, the self-consumption had to be higher than 30% of the electricity produced in the cogeneration plant [41]; in 2004 this limit was reduced to 10% [42]; and in 2006 this restriction was eliminated [43]. Only the surplus produced electricity can be sold to the electric grid.

This section applied the aforementioned different values (corresponding to the legal restrictions on self-consumption of electricity) to the economic optimization model, yielding three scenarios (SC30, SC10, and SC0), shown in Table 9. The scenario SC0, column in bold, corresponds to the base case.

As the real self-consumption of scenario SC0 was 23.83%, the same configuration and operation was maintained when the obligation of self-consumption was raised to 10% in Scenario SC10. However, a slightly different configuration was obtained in Scenario SC30. The obligation of a minimum electricity self-consumption of 30% affected significantly the amount of electricity sold to the electric grid (Scenario SC30: 6620 MWh/yr versus scenarios SC0 and SC10: 11,389 MWh/yr), installing one less gas engine and one more hot water boiler.

The obligation of self-consumption of a portion of the electricity produced by the owner of the cogeneration plant has been a strong restriction in the configuration and operational strategies corresponding to the optimal design. In trigeneration systems, part of the self-consumption could be justified by the operation of mechanical chillers. In fact, this condition limits the quantity of cogeneration modules and absorption chillers to install. The self-consumption obligation has been a persistent barrier to a wider uptake of cogeneration in the residential Spanish sector [21]. As can be seen, the installation of energy-efficient technologies (cogeneration modules and absorption chillers) was fomented by the most recent legal scenario, in which all electricity produced by cogeneration modules can be sold to the electric grid. Various gas engine cogeneration modules were combined with both absorption and compression chillers to satisfy cooling loads.

The right column of Table 9 shows the optimal operation of the base system when an electricity self-consumption of 30% is imposed. The annual total cost obtained (616,644 €) is logically greater than that corresponding to Scenario SC30 with ideal flexible configuration (580,839 €). However that base cost (616,644 €) is significantly lower than the annual total cost of the conventional configuration presented in Table 2 (804,184 €). Again, the base system displayed a desirable robustness feature, being able to adapt to a reasonable variation in the obligatory minimum self-consumption of cogenerated electricity.

### 5.2. Time-of-Delivery Ratio

Time-of-delivery feed-in tariffs help create a more efficient electricity system while providing a means to encourage peak shaving and this can create a number of benefits for electricity customers, grid operators, and society. Some countries provide higher payment levels to encourage electricity generation at times of high demand. Because electricity is more valuable during these times, an incentive structure is one way of aligning the feed-in tariff payment to be more market-oriented [44]. From an investor's point of view, optional hourly differentiation in feed-in tariffs could increases the economic performance and guarantees a secure and predictable cash flow over a determined time period. This contributes to reducing risks and improving returns.

Previous optimizations were carried out considering constantly the price of electricity feed-in to the grid (*p*_es_ = 0.077 €/kWh). The time-of-delivery factor reflected the fact that electricity delivered to the grid during peak times was more valuable than electricity delivered during other times. The Royal Decree 661/2007 [45] divides the day into two periods: 16 on-peak hours (8 h to 24 h) with an increase in price and the 8 remaining off-peak hours (0 h to 8 h) with a discount. Final feed-in electricity price was calculated in function of the time-of-delivery ratio (TDR) solving the following equations:

(6) $$\begin{array}{c} \frac{\left(\text{On-peak}\;\text{price}\right)}{\left(\text{Off-peak}\;\text{price}\right)}=\text{TDR},\\ \left(\text{On-peak}\;\text{price}\right)\cdot 16+\left(\text{Off-peak}\;\text{price}\right)\cdot 8=p_{\text{es}}\cdot 24. \end{array}$$

Data utilized in the optimization model until now did not account for time of delivery and therefore the TDR is 1.0. Ratios of 1.5 and 2.0 were chosen to carry out the sensitivity analyses, and Table 10 shows the obtained results. The scenario SR0, column in bold, corresponds to the base case.

Scenario SR15 presented the same configuration as the base case with the addition of one cooling tower. Operation changed throughout the day to adapt to delivering electricity to the grid at on-peak times. With the implementation of hourly differentiation, no purchase of electricity from the grid occurred. Interestingly, no significant increase in the sale of electricity was verified. Scenario SR20 presented a slight increase in the sale of cogenerated electricity, taking advantage of the higher relationship between on-peak and off-peak prices to realize profit. However, the initial investment in equipment was considerably higher, installing one more gas engine, one less hot water boiler, and switching one mechanical chiller for an absorption chiller.

Only with an exceptional increase in time-of-delivery ratio there was a change in configuration, and the operation of the system did not change significantly. The installation of a thermal energy storage system could change these results: the system would be more flexible and could accumulate cogenerated thermal energy during a time period, to be consumed in other.

In the right column of Table 10 the optimal operation of the base system configuration with a TDR equal to 2.0 is shown. The annual total cost obtained (550,282 €) is slightly higher than that corresponding to Scenario SR20 with ideal flexible configuration (532,662 €), an even lower than the annual total cost corresponding to Scenario SR0 (570,169 €). Again, the base system displayed a desirable robustness feature, being able to adapt to a reasonable variation in the time-of-delivery ratio. Also note that the base system even takes advantage of time-of-delivery feed-in tariff.

## 6. Conclusions

Several sensitivity analyses were carried out to verify the most influential factors on the structure and operation of a trigeneration system designed for a medium-sized hospital.

The terms feasibility, flexibility, robustness, and resilience were defined in the context of synthesis of energy supply systems for buildings. Feasibility concerns about the system (both reference system and trigeneration system) ability to ensure the energy demands of the consumer center. Flexibility took the definition of feasibility a step further, expressing that the system adapted well, economically and efficiently, to expected external changes. The concept of flexibility expressed an upgrade from the reference system (capable to meet the variable energy demands) to the integrated trigeneration system (able to operate efficiently in different expected external conditions). The concept of robustness expressed that the configuration not only fulfilled the expected external conditions in an economic and efficient way, but in the event of unexpected changes, the system adapts well and still delivered an economic and efficient operation. The term resilience expressed the ability of the system to withstand expected changes (flexibility) as well as unexpected changes (robustness).

In the technical sensitivity analyses, as the energy demands decreased, the number of cogeneration modules and the purchase of natural gas decreased. In a resilience analysis considering the variation of energy demands by the consumer center, it was verified that the configuration corresponding to the economic optimal trigeneration system (base system) could absorb variations within the range of −15% and +5%, presenting a performance significantly close to the ideal solution (with free configuration).

In the economic sensitivity analyses, as the amortization and maintenance factor increases, the number of cogeneration modules and absorption chillers decrease along with a decrease in the sale of electricity. An increase in the price of natural gas decreases the benefits achieved, selling less and less electricity and installing less cogeneration modules and absorption chillers. In a resilience analysis considering economic parameters, it was verified that the configuration corresponding to the base system is a satisfactory selection, being stable for a wide interval of amortization factors and natural gas prices, with a behavior very close to the ideal solution.

The obligation of a minimum self-consumption of 30% limited significantly the amount of electricity produced and therefore also the sale of electricity, which confirms that this requirement has been a legal barrier to the penetration of cogeneration in the residential-commercial sector. A change in configuration will only be necessary if there is a significant increase in the time-of-delivery ratio, when the system will realize profit as a consequence of the hourly differentiation in feed-in tariffs.

## Figures and Tables

**Table 1:** Selected equipment and matrix of production coefficients.

Technology *i*	Selected equipment		Utility *j*					
	Cost ZI (€)	Power *P*_nom⁡_ (MW)	EE	CG	WH	WR	WC	AA
Gas engines MGWH	500 250	0.58	**+1**	−2.45	+0.96	+0.20		
Hot water boilers CGWC	34 500	0.57		−1.08	**+1**			
Heat exchangers ICWH	7 475	0.40			−1.00	**+1**		
Absorption chillers FAWH	230 000	0.49	−0.01		−1.50	+2.50	**+1**	
Mechanical chillers FMWR	201 250	0.49	−0.23			+1.23	**+1**	
Cooling towers CTWR	28 750	1.00	−0.02			−1.00		**+1**

**Table 2:** Results for optimal trigeneration system (base case) and conventional system.

System composition	Conventional system		Trigeneration system	
	Number	Total power	Number	Total power
Gas engines	—	—	3	1739 kW
Hot water boilers	6	3420 kW	3	1710 kW
Heat exchangers	—	—	4	1600 kW
Absorption chillers	—	—	1	490 kW
Mechanical chillers	4	1960 kW	3	1470 kW
Cooling towers	3	3000 kW	3	3000 kW

Natural gas (total) MWh/yr	8703		37,324	
Purchased electricity MWh/yr	3572		29	
Sold electricity MWh/yr	—		11,389	
Natural gas (cogeneration) MWh/yr	—		36,638	
Cogenerated electricity MWh/yr	—		14,954	
Cogenerated useful heat MWh/yr	—		8,602	

Primary energy savings %	—		10.01	
Equivalent electrical efficiency %	—		55.22	

*Annual fixed cost €/yr *	*219,650 *		*510,830 *	
Cost of natural gas €/yr	217,582		933,092	
Cost of electricity €/yr	366,951		3207	
Profit with the sale of electricity €/yr	—		−876,960	
*Annual energy cost €/yr *	*584,534 *		*59,339 *	

Annual total cost €/yr	804,184		570,169	

**Table 3:** Sensitivity analysis for a variation in energy demands.

Variation	−20%	−10%	**0%**	+10%	+20%
System composition					
Gas engines	2	2	**3**	3	3
Hot water boilers	3	3	**3**	3	4
Heat exchangers	3	3	**4**	4	4
Absorption chillers	1	1	**1**	1	1
Mechanical chillers	2	3	**3**	3	4
Cooling towers	3	3	**3**	4	4

Natural gas (total) MWh/yr	25,896	26,349	**37,324**	38,301	38,689
Purchased electricity MWh/yr	0	9	**29**	0	0
Sold electricity MWh/yr	7340	6981	**11,389**	11,269	10,905
Natural gas (cogeneration) MWh/yr	24,896	24,831	**36,638**	37,246	37,189
Cogenerated electricity MWh/yr	10,162	10,135	**14,954**	15,202	15,179
Cogenerated useful heat MWh/yr	6598	6981	**8602**	9161	9609

Primary energy savings %	12.65	13.99	10.01	11.00	12.08
Equivalent electrical efficiency %	57.85	59.36	**55.22**	56.17	57.25

Annual fixed cost €/yr	369,035	409,285	**510,830**	516,580	563,730
Cost of natural gas €/yr	647,391	658,718	**933,092**	957,513	967,215
Cost of electricity €/yr	0	1076	**3207**	0	0
Profit with the sale of electricity €/yr	−565,160	−537,535	**−876,960**	−867,692	−839,669

Annual total cost €/yr	451,266	531,544	**570,169**	606,401	691,275

**Table 4:** Sensitivity analysis for variations in energy demands considering the base case configuration.

Variation	−20%	−15%	−10%	−5%	**0%**	+5%
System composition						
Gas engines	3	3	3	3	**3**	3
Hot water boilers	3	3	3	3	**3**	3
Heat exchangers	4	4	4	4	**4**	4
Absorption chillers	1	1	1	1	**1**	1
Mechanical chillers	3	3	3	3	**3**	3
Cooling towers	3	3	3	3	**3**	3

Natural gas (total) MWh/yr	31,921	33,520	34,976	36,407	**37,324**	37,389
Purchased electricity MWh/yr	0	0	9	16	**29**	45
Sold electricity MWh/yr	10,090	10,521	10,892	11,244	**11,389**	11,188
Natural gas (cogeneration) MWh/yr	31,757	33,256	34,589	35,880	**36,638**	36,520
Cogenerated electricity MWh/yr	12,962	13,574	14,118	14,645	**14,954**	14,906
Cogenerated useful heat MWh/yr	7371	7719	8028	8328	**8602**	8857

Primary energy savings %	9.77	9.77	9.77	9.77	**10.01**	10.70
Equivalent electrical efficiency %	55.00	55.00	55.00	55.00	**55.22**	55.87

Annual fixed cost €/yr	510,830	510,830	510,830	510,830	**510,830**	510,830
Cost of natural gas €/yr	798,035	838,003	874,395	910,169	**933,092**	934,714
Cost of electricity €/yr	0	0	1076	1720	**3207**	5083
Profit with the sale of electricity €/yr	−776,967	−810,149	−838,713	−865,824	**−876,960**	−861,441

Annual total cost €/yr	531,899	538,684	547,588	556,895	**570,169**	589,186

**Table 5:** Sensitivity analysis for fam factor.

fam (yr^−1^)	0.10	0.15	**0.20**	0.25	0.30
System composition					
Gas engines	3	3	**3**	2	2
Hot water boilers	3	3	**3**	4	4
Heat exchangers	4	4	**4**	3	3
Absorption chillers	2	2	**1**	1	1
Mechanical chillers	2	2	**3**	3	3
Cooling towers	4	4	**3**	3	3

Natural gas (total) MWh/yr	38,028	38,028	**37,324**	26,847	26,847
Purchased electricity MWh/yr	0	0	**29**	29	29
Sold electricity MWh/yr	11,712	11,712	**11,389**	6620	6620
Natural gas (cogeneration) MWh/yr	37,344	37,344	**36,638**	24,741	24,741
Cogenerated electricity MWh/yr	15,242	15,242	**14,954**	10,098	10,098
Cogenerated useful heat MWh/yr	9075	9075	**8602**	7288	7288

Primary energy savings %	10.74	10.74	10.01	15.08	15.08
Equivalent electrical efficiency %	55.91	55.91	**55.22**	60.68	60.68

Annual fixed cost €/yr	261,165	391,747	**510,830**	520,231	624,278
Cost of natural gas €/yr	950,705	950,705	**933,092**	671,163	671,163
Cost of electricity €/yr	0	0	**3207**	3207	3207
Profit with the sale of electricity €/yr	−901,838	−901,838	**−876,960**	−509,717	−509,717

Annual total cost €/yr	310,032	440,614	**570,169**	684,885	788,931

**Table 6:** Sensitivity analysis for fam considering the base case configuration.

fam (yr^−1^)	0.10	0.15	**0.20**	0.25	0.30
System composition					
Gas engines	3	3	**3**	3	3
Hot water boilers	3	3	**3**	3	3
Heat exchangers	4	4	**4**	4	4
Absorption chillers	1	1	**1**	1	1
Mechanical chillers	3	3	**3**	3	3
Cooling towers	3	3	**3**	3	3

Natural gas (total) MWh/yr	37,324	37,324	**37,324**	37,324	37,324
Purchased electricity MWh/yr	29	29	**29**	29	29
Sold electricity MWh/yr	11,389	11,389	**11,389**	11,389	11,389
Natural gas (cogeneration) MWh/yr	36,638	36,638	**36,638**	36,638	36,638
Cogenerated electricity MWh/yr	14,954	14,954	**14,954**	14,954	14,954
Cogenerated useful heat MWh/yr	8602	8602	**8602**	8602	8602

Primary energy savings %	10.01	10.01	**10.01**	10.01	10.01
Equivalent electrical efficiency %	55.22	55.22	**55.22**	55.22	55.22

Annual fixed cost €/yr	255,415	383,122	**510,830**	638,538	766,245
Cost of natural gas €/yr	933,092	933,092	**933,092**	933,092	933,092
Cost of electricity €/yr	3207	3207	**3207**	3207	3207
Profit with the sale of electricity €/yr	−876,960	−876,960	**−876,960**	−876,960	−876,960

Annual total cost €/yr	314,754	442,462	**570,169**	697,877	825,584

**Table 7:** Sensitivity analysis for natural gas prices.

*p*_*g*_ (€/kWh)	0.015	0.020	**0.025**	0.030	0.035
System composition					
Gas engines	3	3	**3**	2	1
Hot water boilers	3	3	**3**	4	5
Heat exchangers	4	4	**4**	2	1
Absorption chillers	2	2	**1**	1	1
Mechanical chillers	2	2	**3**	3	3
Cooling towers	4	4	**3**	3	3

Natural gas (total) MWh/yr	38,028	38,028	**37,324**	25,977	17,199
Purchased electricity MWh/yr	0	0	**29**	29	83
Sold electricity MWh/yr	11,712	11,712	**11,389**	6273	1660
Natural gas (cogeneration) MWh/yr	37,344	37,344	**36,638**	23,871	12,425
Cogenerated electricity MWh/yr	15,242	15,242	**14,954**	9743	5072
Cogenerated useful heat MWh/yr	9075	9075	**8602**	7288	4525

Primary energy savings %	10.74	10.74	10.01	15.93	20.32
Equivalent electrical efficiency %	55.91	55.91	**55.22**	61.77	68.55

Annual fixed cost €/yr	522,330	522,330	**510,830**	414,690	320,045
Cost of natural gas €/yr	570,423	760,564	**933,092**	779,306	599,155
Cost of electricity €/yr	0	0	**3207**	3207	9424
Profit with the sale of electricity €/yr	−901,838	−901,838	**−876,960**	−483,019	−127,847

Annual total cost €/yr	190,915	381,056	**570,169**	714,185	800,776

**Table 8:** Sensitivity analysis for natural gas price considering the base case configuration.

*p*_*g*_ (€/kWh)	0.015	0.020	**0.025**	0.030	0.035
System composition					
Gas engines	3	3	**3**	3	3
Hot water boilers	3	3	**3**	3	3
Heat exchangers	4	4	**4**	3	1
Absorption chillers	1	1	**1**	1	1
Mechanical chillers	3	3	**3**	3	3
Cooling towers	3	3	**3**	3	3

Natural gas (total) MWh/yr	37,338	37,324	**37,324**	37,324	24,218
Purchased electricity MWh/yr	27	29	**29**	29	34
Sold electricity MWh/yr	11,389	11,389	**11,389**	11,389	6089
Natural gas (cogeneration) MWh/yr	36,638	36,638	**36,638**	36,638	23,437
Cogenerated electricity MWh/yr	14,954	14,954	**14,954**	14,954	9566
Cogenerated useful heat MWh/yr	8602	8602	**8602**	8602	8241

Primary energy savings %	10.01	10.01	**10.01**	10.01	19.42
Equivalent electrical efficiency %	55.22	55.22	**55.22**	55.22	67.00

Annual fixed cost €/yr	510,830	510,830	**510,830**	510,830	510,830
Cost of natural gas €/yr	560,064	746,473	**933,092**	1,119,719	847,624
Cost of electricity €/yr	2990	3207	**3207**	3207	3731
Profit with the sale of electricity €/yr	−876,960	−876,960	**−876,960**	−876,960	−468,824

Annual total cost €/yr	196,924	383,551	**570,169**	756,787	893,361

**Table 9:** Sensitivity analyses for legal constraints on mandatory self-consumption.

Scenario	**SC0**	SC10	SC30	BASE
Obligation of self-consumption	**0%**	>10%	>30%	>30%
System composition				
Gas engines	**3**	3	2	3
Hot water boilers	**3**	3	4	3
Heat exchangers	**4**	4	3	4
Absorption chillers	**1**	1	1	1
Mechanical chillers	**3**	3	3	3
Cooling towers	**3**	3	3	3

Natural gas (total) MWh/yr	**37,324**	37,324	26,847	29,171
Purchased electricity MWh/yr	**29**	29	29	29
Sold electricity MWh/yr	**11,389**	11,389	6620	8139
Natural gas (cogeneration) MWh/yr	**36,638**	36,638	24,741	28,485
Cogenerated electricity MWh/yr	**14,954**	14,954	10,098	11,627
Cogenerated useful heat MWh/yr	**8602**	8602	7288	8601

Primary energy savings %	**10.01**	10.01	15.08	15.08
Equivalent electrical efficiency %	**55.22 **	55.22	60.68	61.42

Annual fixed cost €/yr	**510,830**	510,830	416,185	510,830
Cost of natural gas €/yr	**933,092**	933,092	671,163	729,285
Cost of electricity €/yr	**3207**	3207	3207	3207
Profit with the sale of electricity €/yr	**−876,960**	−876,960	−509,717	−626,678

Annual total cost €/yr	**570,169**	570,169	580,839	616,644

**Table 10:** Sensitivity analyses for hourly differentiation in the feed-in tariff.

Scenario	**SR0**	SR15	SR20	BASE
Time-of-delivery ratio (TDR)	**1.0**	1.5	2.0	2.0
System composition				
Gas engines	**3**	3	4	**3**
Hot water boilers	**3**	3	2	**3**
Heat exchangers	**4**	4	5	**4**
Absorption chillers	**1**	1	2	**1**
Mechanical chillers	**3**	3	2	**3**
Cooling towers	**3**	4	4	**3**

Natural gas (total) MWh/yr	**37,324**	32,812	39,326	31,235
Purchased electricity MWh/yr	**29**	0	0	29
Sold electricity MWh/yr	**11,389**	9555	12,384	8884
Natural gas (cogeneration) MWh/yr	**36,638**	32,110	39,092	30,384
Cogenerated electricity MWh/yr	**14,954**	13,106	15,931	12,402
Cogenerated useful heat MWh/yr	**8602**	8589	9379	8393

Primary energy savings %	**10.01**	12.86	10.50	13.59
Equivalent electrical efficiency %	**55.22 **	58.10	55.68	58.89

Annual fixed cost €/yr	**510,830**	516,580	616,975	510,830
Cost of natural gas €/yr	**933,092**	820,293	983,159	780,864
Cost of electricity €/yr	**3207**	0	0	3207
Profit with the sale of electricity €/yr	**−876,960**	−768,094	−1,067,473	−744,619

Annual total cost €/yr	**570,169**	568,780	532,662	550,282

## Nomenclature

*C*_fix_: Annual fixed cost of the equipment [€/yr]
*C*_ope_: Annual operational cost (energy) [€/yr]
*C*_tot_: Annual total cost [€/yr]
*d*: Day of the year
EEE: Equivalent electrical efficiency [%]
*E*_*c*_: Cogenerated electricity [MWh/yr]
*E*_*d*_: Electricity demand [MW]
*E*_*p*_: Electricity purchased (imported from grid) [MW]
*E*_*s*_: Electricity sold (exported to the grid) [MW]
fam: Amortization factor [yr^−1^]
*F*_*c*_: Consumption of natural gas (cogeneration) [MWh/yr]
*F*_*g*_: Consumption of natural gas [MW]
*h*: Hour of the day
*i*: Index refers to technology *i*
NIN(*i*): Number of pieces of equipment (technology *i*) installed
PES: Primary energy savings [%]
*p*_ep_: Price of purchased electricity [€/MWh]
*p*_es_: Price of sold electricity [€/MWh]
*p*_*g*_: Price of natural gas [€/MWh]
*P*_nom⁡_: Nominal power [MW]
*Q*_cc_: Cogenerated useful heat [MWh/yr]
*Q*_*d*_: Heat demand [MW]
*R*_*d*_: Cooling demand [MW]
TDR: Time-of-delivery ratio
*Z*(*i*): Total investment cost [€] of technology *i*
ZI(*i*): Capital cost [€] of each piece of equipment installed for technology *i*.

### Greek Letters

*η*_ec_: Efficiency value for separate production of electricity
*η*_qc_: Efficiency value for separate production of heat.
